# High persistence of biologic therapy in patients with Psoriatic arthritis: a real-world evidence from a high-complexity hospital in Colombia

**DOI:** 10.3389/fphar.2025.1559168

**Published:** 2025-08-29

**Authors:** Andrés Hormaza-Jaramillo, Leidy Johanna Hurtado-Bermudez, Daniela Peñaloza Gonzalez, Tatiana Delgado-Mora

**Affiliations:** ^1^ Fundación Valle del Lili, Centro de Investigaciones Clínicas, Cali, Colombia; ^2^ Fundación Valle del Lili, Department of Internal Medicine, Service of Rheumatology, Cali, Colombia; ^3^ Faculty of Health Sciences, Department of Public Health, Universidad Icesi, Cali, Colombia

**Keywords:** Psoriatic arthritis, biologics, persistence, treatment, Rheumatology

## Abstract

**Introduction:**

Psoriatic arthritis (PsA) is a chronic inflammatory disease that significantly impacts patients’ quality of life, underscoring the importance of timely diagnosis and appropriate treatment. In Colombia, the estimated prevalence is 13.5 cases per100,000 inhabitants; however, few studies have addressed this condition in the local context. Although there is no single international consensus on treatment, various clinical guidelines agree on the effectiveness of biologic therapies. Despite this, data on Colombian patients treated with biologic agents remain scarce. Therefore, this study aims to describe the clinical and paraclinical characteristics, as well as the outcomes, of patients with PsA receiving biologic treatments—representing the first such analysis conducted in our country.

**Methods:**

A retrospective descriptive study was conducted using medical records of patients with PsA treated with biologic therapies at a high-complexity hospital in Colombia between 2011 and 2021.

**Results:**

A total of 127 patients were included, 55.1% of whom were women, with a mean age of 50.3 years. Hypertension was the most common comorbidity, and peripheral arthritis was the most frequent subtype (55.7%). TNF inhibitors were the most commonly used biologics, followed by IL-17 and IL-12/23 inhibitors, with greater persistence observed with secukinumab and adalimumab. A total of 39.4% of patients switched biologics, most commonly to secukinumab. Only 5.5% discontinued treatment, primarily due to infections. The median time in biological therapy was 36.5 months.

**Conclusion:**

Few studies described PsA patients treated with biologics in Latin America, making these findings a valuable contribution on biologic usage and persistence patterns in Colombia, with a high persistence population. The results highlight the heterogeneity of this disease and the need for personalized, continuous management guided by specialists.

## Introduction

Psoriatic arthritis (PsA) is a chronic, progressive, inflammatory, and heterogeneous disease that often becomes degenerative and challenging to diagnose, significantly impacting patients’ quality of life and posing public health concerns (López-Ferrer and Laiz-Alonso, 2014; Cantini et al., 2010; Ogdie et al., 2020).

Estimates of PsA prevalence at both global and continental levels are derived from epidemiological studies that employ diverse methodologies. In these studies, PsA was identified through clinical diagnosis or internationally recognized classification criteria, such as Classification Criteria for Psoriatic Arthritis (CASPAR) (Veale et al., 2025). However, diagnosing PsA can be complex and requires comprehensive clinical evaluation, including physical examination, patient-reported symptoms, and imaging or laboratory tests (Lembke et al., 2024).

Scientific literature indicates that between 14% and 30% of patients with psoriasis develop PsA, which is associated with worse functional status and greater disability compared to patients with psoriasis without PsA (Ogdie et al., 2020; Kamata et al., 2020; Elalouf and Chandran, 2018; Fernández-Ávila et al., 2023a). According to data from Colombia’s national health registry, the prevalence of PsA between 2012 and 2018 was 13.5 cases per 100,000 inhabitants (Fernández-Ávila et al., 2023b). Furthermore, Colombian patients with PsA have been reported to experience significantly worse quality-of-life scores, higher absenteeism, and greater work-related impact compared to patients with psoriasis without PsA (Fernández-Ávila et al., 2023a).

The existing guidelines such as EULAR/ACR/GRAPPA for PsA cover many aspects of management. Some gaps remain relating to routine practice application. (Coates et al., 2025). Despite this, most evidence-based literature agrees that treatment with biologics is an effective approach for managing PsA. However, there is considerable variability in clinical responses to biological therapies among individuals, possibly due to the heterogeneity of clinical presentations, broad genotypic and phenotypic variability, and differences in serum drug concentrations across populations and dosing regimens (Ruyssen-Witrand et al., 2020). Additionally, the development of anti-drug antibodies following the administration of these therapies can reduce therapeutic response by up to 80% (Ruyssen-Witrand et al., 2020).

In the Latin American context, optimal management of PsA patients remains a significant challenge, with an increasing number of available pharmacological options, including a considerable group of biologic drugs (Fernández-Ávila et al., 2023a). Overall, data on biologic therapy use patterns in PsA patients remains limited in Colombia (Walsh et al., 2018). Therefore, this study aims to describe the demographic and clinical characteristics of PsA patients treated with biologic drugs at a high-complexity institution in Colombia. Additionally, the study seeks to identify the most frequently administered medications and their usage patterns.

This study was conducted between 2011 and 2021, including only medications approved by the Colombian National Institute for Food and Drug Surveillance (INVIMA) during this period. Consequently, recently approved biologic therapies such as JAK inhibitors and other drugs for PsA management are not discussed (República de Colombia and Ministerio de Salud y Protección SocialInstituto Nacional de Vigi lancia de Medicamentos y Alimentos–INVIMA).

## Materials and methods

Study design: Observational longitudinal descriptive study with retrospective data collection.

### Selection criteria

Inclusion: Patients aged 18 years and older who were treated at Fundación Valle del Lili between 2011 and 2021 and had a diagnosis of PsA, identified using the following ICD-10 codes: M07.0, M07.2, M07.3, M09.0, and L40.5, and patients with diagnosis of PsA confirmed by a rheumatologist, in accordance with the CASPAR classification criteria (This included patients with psoriasis or inflammatory bowel disease (IBD), patients who have had previous biological therapy or who have not had a complete follow up if they met the diagnostic criteria for PsA).

Exclusion: Patients receiving biologic therapy for other conditions without a confirmed PsA diagnosis.

Data Collection: For each patient, clinical records were reviewed once, retrospectively, covering three time points: baseline (study inclusion), 12 months, and 24 months. The selection of these time points was based on standard clinical follow-up intervals commonly used in the management of patients receiving biological therapy. This approach allowed for a reliable assessment of treatment persistence and therapy changes over time. However, not all patients had follow-up data at the 2-year mark, since not all of them had further consultations in the institution. Patient data were anonymized by assigning unique numeric codes prior to analysis. No identifiable information was retained in the working databases.

Sample Size and Sampling Strategy: A non-probabilistic, non-sequential convenience sampling method was used, including 127 patients who met the selection criteria. The list of patients who met the inclusion criteria was provided by the Statistics Department of the institution. Subsequently, the medical records were reviewed and, data corresponding to the variables of interest was extracted and entered into the study database for analysis.

Variables: To characterize the patients and meet the study’s objectives, we analyzed the following:

• Demographics: Sex and age.
• Personal and family history: Any autoimmune or autoinflammatory disease that the patient or their family members have experienced previously. This includes conditions such as hypothyroidism, systemic lupus erythematosus (SLE), and psoriasis.
• Psoriatic arthritis subclassification: peripheral, mixed, no evidence of joint activity and axial spondylitis and/or sacroilitis) categorized according to joint involvement.
o Mixed arthritis: Refers to the coexistence of peripheral arthritis and axial confirmed by imaging.
o Enthesitis was recorded based on clinical examination, but no validated enthesitis scoring system was systematically applied.
• Psoriasis characterization: History, subtype, and whether it was present at study entry.
• Paraclinical markers: Paraclinical findings encompass laboratory markers such as erythrocyte sedimentation rate (ESR), C-reactive protein (PCR), hemoglobin, leukocyte count, platelet count and lymphocyte count.
• Biologic drug usage: Most used biologics and their patterns (type, duration, switches).
• Therapies: Biologic and non-biologic treatments; previous therapies and alternatives.
• Imaging Diagnostics: Magnetic Resonance Imaging (MRI) (sacroiliitis, synovitis, enthesitis, erosions, new bone formation, periostitis), X-ray spinal (erosions, periostitis, syndesmophytes, new bone formation, Initial hand) X-ray (pencil-in-cup deformities, acroosteolysis, erosions, periostitis) and Follow-up (hand X-ray persistence or progression of the above findings).
• Complications: These were classified as relevant comorbidities and included prior coronary artery bypass surgery, myocardial ischemia with aortic aneurysm, and one case of coronary artery disease occurring after therapy initiation.

Statistical Analysis: Descriptive statistical analysis was performed, summarizing qualitative variables using frequencies and percentages, and quantitative variables using means and standard deviations or medians and interquartile ranges, depending on variable distribution, assessed with the Kolmogorov-Smirnov normality test. Some data were visually summarized using graphs to enhance result interpretation. Descriptive analyses were conducted using Stata version 16.

Ethical Considerations: This study was approved by the institutional ethics committee of the Fundación Valle del Lili University Hospital in Cali, Colombia (Approval # 539).

## Results

A total of 127 patients were analyzed. Women represented 55.1% of the cohort, with a mean age of 50.3 years. A history of autoimmune or autoinflammatory diseases was present in 93.7% of patients, with psoriasis being the most common (94.1%). The most frequent comorbidity was hypertension (50.6%), and osteoarthritis (8.2%). Additionally, 20.5% had a family history of autoimmune diseases (Table 1).

**TABLE 1:** Demographic, clinical and paraclinical characteristics of patients with psoriatic arthritis managed with biological therapy.

Variable	N = 127	n (%)
Sociodemographic Characteristics	127	
Female gender		70 (55.1)
Age in years*		50.3 (13.3)
Personal History	127	
History of autoimmune/autoinflammatory disease		119 (93.7)
Autoimmune comorbidities	119	
Psoriasis		112 (94.1)
Hypothyroidism		3 (2.5)
Non-autoimmune disease history	127	85 (66.9)
Non-autoimmune comorbidities	85	
Hypertension		43 (50.6)
Osteoarthritis		7 (8.2)
Dyslipidemia		3 (3.5)
Overweigh/Obesity		3 (3.5)
Family history	127	
Autoimmune disease history		
Autoimmune comorbidities (family)	127	26 (20.5)
Psoriasis		8 (6.3)
Rheumatoid arthritis		6 (4.7)
Psoriatic arthritis subclassification	122	
Psoriatic arthritis subclassification		
Peripheral arthritis		68 (55.7)
Mixed arthritis (peripheral and axial)		31 (25.4)
No evidence of joint activity		13 (10.7)
Axial spondylitis and/or sacroiliitis		10 (8.2)
Type of joint involvement	127	
Symmetrical joint involvement		57 (44.9)
Joint tenderness		39 (30.7)
Asymmetrical joint involvement		37 (29.1)
Joint deformity		10 (7.9)
Local joint warmth		8 (6.3)
Clinical manifestations	127	
Joint pain		105 (82.7)
Low back pain		39 (30.7)
Enthesitis		33 (26.0)
Morning stiffness		28 (22.0)
Joint swelling		26 (20.5)
Dactylitis		25 (19.7)
Movement limitation		23 (18.1)
Sacroiliitis		23 (18.1)
Synovitis		19 (15.0)
Heel pain		19 (15.0)
Inflammatory neck pain		16 (12.6)
Psoriasis characterization		
History of psoriasis	127	113 (89.0)
Current psoriasis	127	98 (77.1)
Time to onset of psoriatic arthritis**	39	60 (127)
Psoriasis subtype	113	
Vulgar psoriasis		44 (39.0)
Guttate psoriasis		8 (7.0)
Nail psoriasis		7 (6.2)
Type of lesions	113	
Other lesions		31 (27.4)
Scalp lesions		30 (26.5)
Nail dystrophy		22 (19.4)
No lesions		8 (7.1)
Diagnostic Aids		
Elevated ESR	127	31 (24.4)
HLA-B27 positive	127	11 (8.7)
Rheumatoid factor positive	127	5 (3.9)
Positive ANA	127	8 (6.3)
ANA titer value**	8	240 (160)
Laboratory Results		
Leukocyte count*	110	7373.3 (2179.5)
Hemoglobin*	107	14.2 (1.6)
Platelet count*	104	279990.4 (79910)
Lymphocyte count*	85	2332.4 (886.9)
PCR level**	69	0.56 (1.1)
Biological Screening		
Positive tuberculin test	127	16 (12.6)
Elevated ALT	127	16 (12.6)
Elevated AST	127	12 (9.5)
Elevated alkaline phosphatase	127	7 (5.5)
Imaging Diagnostics		
Magnetic Resonance Imaging	127	34 (26.8)
X-ray spinal	127	18 (14.2)
Initial hand X-ray	127	19 (15.0)
Follow-up hand X-ray	19	14 (73.7)
Complications	127	
ICU management		2 (1.6)
Cardiovascular alterations		3 (2.4)

Source: Author´s own elaboration.

* Mean (Standard Deviation) ** Median (Interquartile Range).

The most common type of arthritis was peripheral arthritis (55.7%), followed by mixed arthritis (25.4%). The most frequently reported symptom was joint pain (82.7%), with symmetric joint involvement in 44.9% of cases and asymmetric involvement in 29.1%. Other reported symptoms included low back pain (30.7%) and enthesitis (26.0%), and morning stiffness (22.0%).

Among laboratory findings, elevated ESR was the most frequent abnormal marker (24.4%). Spinal MRI showed the highest rate of imaging abnormalities (26.8%). None of the cardiovascular alterations reported (2.4%) were considered treatment-related adverse events. Only 1.6% of patients required intensive care unit (ICU) management (Table 1). Only one of the patients admitted to the ICU was due to therapy related complications.

Regarding treatment persistence, only 5.5% of the patients discontinued biological therapy without switching or restarting it, while 12.6% temporarily discontinued the therapy and later resumed it. The median duration of continuous biological therapy without interruptions was 36.5 (IQR = 50.0) months. The main reason for discontinuation was adverse effects (34.8%), followed by clinical improvement (17.4%) and other reasons (3.5%), as detailed in Table 2.

**TABLE 2:** Discontinuation of biological therapy.

Variable	N = 127	n (%)
Biological Treatment	127	
Time on biological therapy (months)**	88	36.5 (50.0)
Required discontinuation of biological therapy	127	
Partial suspension		16 (12.6)
Total suspension		7 (5.5)
Reasons for discontinuation of biological	23	
Adverse effect		8 (34.8)
Clinical improvement		4 (17.4)
Other		4 (17.4)
Administrative procedures		3 (13.0)
Patient´s decision		1 (4.3)

Source: Author´s own elaboration.

* Mean (Standard Deviation) ** Median (Interquartile Range).

The most prescribed complementary therapies were conventional DMARDs (57.5%), methotrexate (44.1%), corticosteroids (27.6%), and nonsteroidal anti-inflammatory drugs (NSAIDs) (22.8%). In terms of therapy changes, 60.6% remained on their initial biologic treatment, whereas 39.4% switched to a different biologic agent. This pattern reflects a common clinical strategy of switching the mechanism of action (MOA) when treatment efficacy is suboptimal. Additionally, patient distribution is reported according to the biological agent administered at each treatment change, with secukinumab being the final agent in 31.7% of cases (Supplementary Material 1). The main reason for switching was a lack of response to treatment in more than 60% of patients (Supplementary Material 2).

**FIGURE 1:**
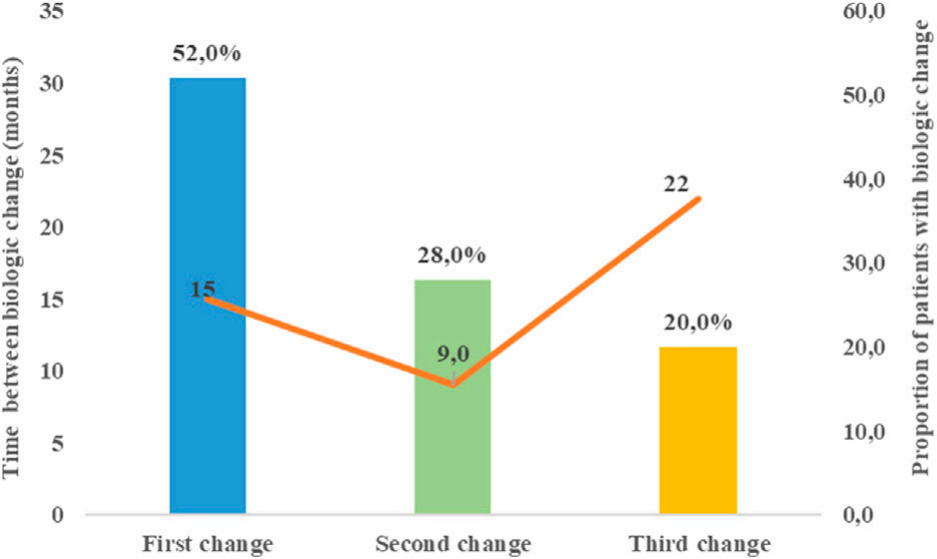
Proportion of patients with a change in biological agent and average time between changes.

Of the 50 patients who experienced a change in their biological therapy, 52.0% had one change, 28.0% had two changes, and 20% had three changes. The median time between the initiation of biological therapy and the first change was 15 (IQR = 18.9) months, between the first and second biological agent was 9 (IQR = 9.0) months, and between the second and third was 22 (IQR = 43.0) months Figure 1.

**FIGURE 2:**
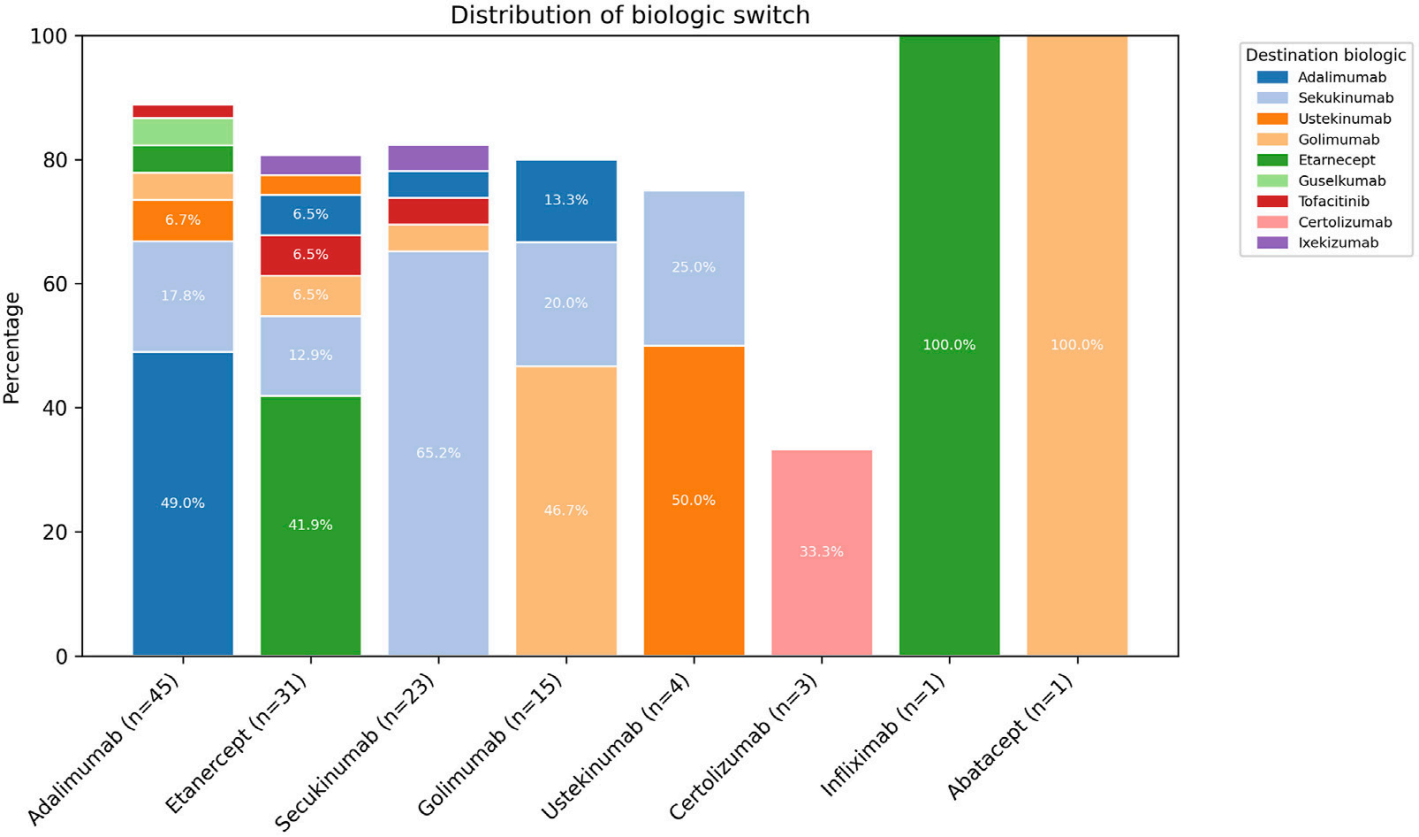
Initial and final biological agents in patients with psoriatic arthritis.

The four most commonly used biological agents to initiate treatment were adalimumab (35.4%), etanercept (24.4%), secukinumab (18.1%), and golimumab (11.8%). Approximately half of the patients who started with adalimumab continued with the same biological agent (49.0%); for etanercept, this proportion was 41.9%; for secukinumab, it was 65.2%; and for golimumab, it was 46.7%. Among these four biological agents, the most common change option was secukinumab (Figure 2).

**FIGURE 3:**
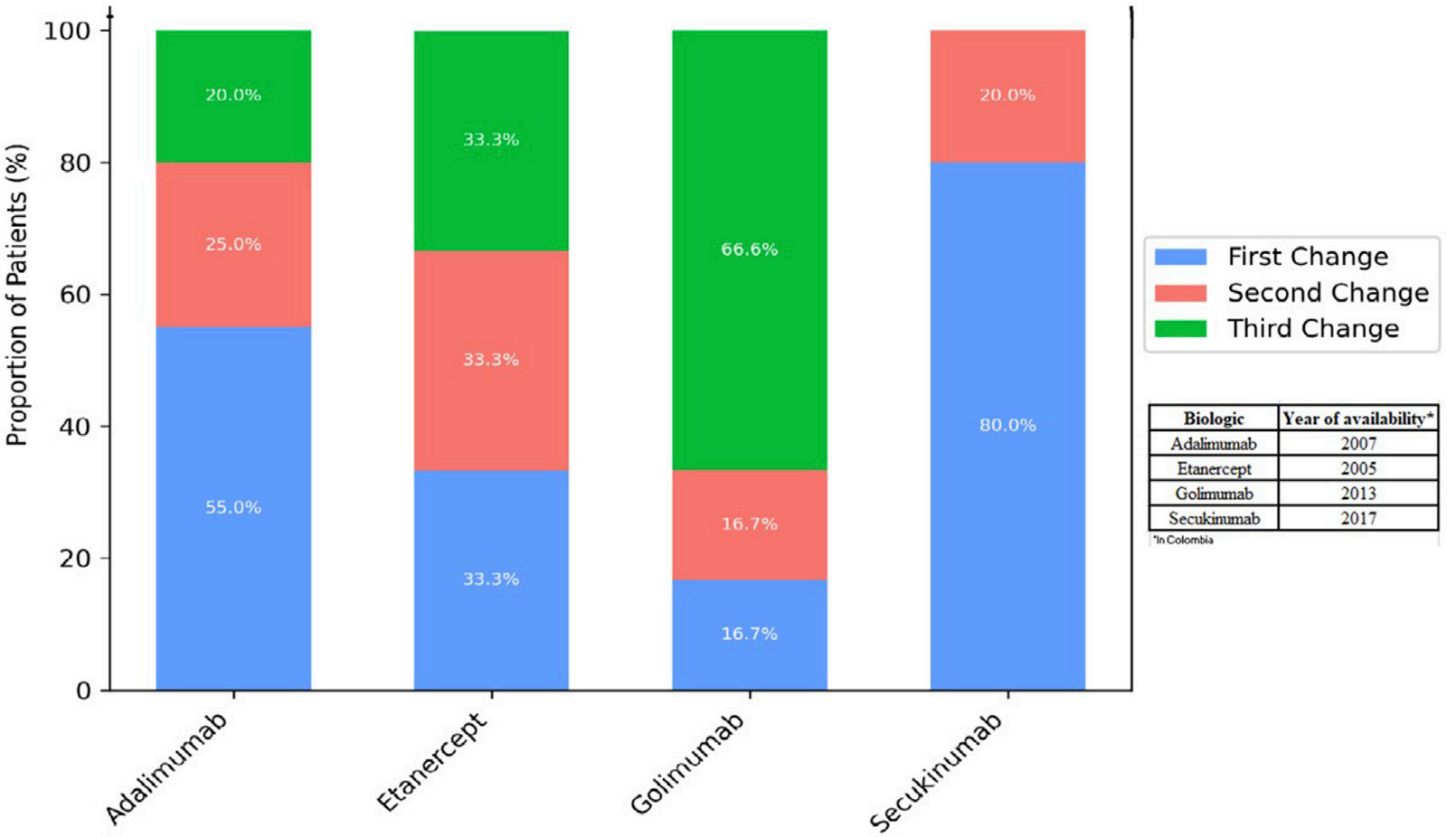
Number of changes by most commonly administered biological agent.

The percentage of individuals with a change in the four most commonly administered biological agents at the start of therapy is shown in Figure 3. Of the 50 individuals who experienced a change, 40% were on TNF inhibitor - adalimumab, 30% on etanercept, 12% on golimumab, and 10% on secukinumab an IL-17 inhibitor, was the most frequently selected subsequent agent, suggesting that a considerable proportion of treatment changes involved a shift to a different mechanism of action. The highest percentage of changes in each biological agent occurred with the first change, with 55%, 33%, 16%, and 80%, respectively (Figure 3).

## Discussion

The treatment of PsA should be based on an individualized assessment of each patient (Fernández-Ávila et al., 2023a; Merola et al., 2017). Although several management guidelines are available, the variability in PsA’s clinical presentation complicates its diagnosis (Ogdie et al., 2020). Nevertheless, scientific societies agree that the use of biological agents is both safe and effective (Kamata et al., 2020; Ruyssen-Witrand et al., 2020), as they help alleviate symptoms, prevent joint damage, and improve patients’ quality of life and functional status (D’Angelo et al., 2017).

Biologic therapies are gaining increasing importance in PsA management. While extensive global data exist on PsA patients treated with biologics, evidence from Latin America remains limited (Walsh et al., 2018). This is the first study conducted at a high-complexity hospital in Colombia that characterizes PsA patients treated with biologic agents, examining the most frequently used drugs and their usage patterns.

In this descriptive study, a total of 127 patients with PsA who received treatment with biologic agents were analyzed. Of these, 55.1% were female, and the average (Sd) age was 50.3 (13.3) years. In our cohort, the most frequent comorbidity was hypertension (50.6%), followed by osteoarthritis (8.2%) and dyslipidemia (3.5%). Similarly, a registry of PsA patients in the United States reported that, among those receiving biologic therapy, 54% were women, with an average age of 54.7 years, and more than half (62.2%) had some form of cardiovascular disease as a comorbidity (Mease et al., 2018). Additionally, a study on the interaction of PsA with cardiometabolic diseases found that hypertension was the most frequent comorbidity in their cohort (28%) (Lorenzo Martín et al., 2022). These findings are consistent with previous studies reporting a high burden of cardiometabolic conditions in PsA patients.

In Latin America, data on the clinical and sociodemographic characteristics of PsA patients remain limited. To our knowledge, no previous studies have been conducted in Colombia describing these characteristics in patients treated with biologic therapies. However, the overall prevalence of PsA in Colombia, based on national health registry data, was 13.5 cases per 100,000 inhabitants, with a total of 6,433 PsA cases reported between 2012 and 2018 (Fernández-Ávila et al., 2023b).

Regarding the clinical characteristics of patients treated with biologic drugs, this study found that the most frequent clinical subtype of PsA was peripheral arthritis (55.7%), and the most common symptoms were joint pain (82.7%), with a predominance of symmetric (44.9%) and asymmetric (29.1%) involvement, in addition to lower back pain (30.7%) and enthesitis (26%).

In contrast, a registry of PsA patients treated with biologics in the United States found that the most frequent symptoms in their population were morning stiffness (92.5%), enthesitis (31.8%), joint pain (25.3%), and dactylitis (13.5%) (Mease et al., 2018). Studies in Spain and Greece also confirmed a higher prevalence of peripheral arthritis (72.2%–95%) and additional manifestations such as onychopathy (22.9%–45%) and enthesitis (12%–15%) in both countries respectively (Klavdianou et al., 2022; García Porrúa et al., 2021).

These findings highlight the heterogeneity in the clinical presentation of PsA, which may be influenced by population-specific characteristics and methodological differences across studies.

In our study the most used biologics to initiate therapy in PsA patients were TNF inhibitors, such as Adalimumab (35.4%) and Etanercept (24.4%). Followed by IL-17 inhibitors, such as Secukinumab (18.1%), IL-12/23 inhibitors, such as Ustekinumab (3.2%), and IL-23 inhibitors, such as Guselkumab (2.4%). A multicenter study in Argentina showed similar preferences, with Adalimumab (45.8%), and Etanercept (36.1%) being the most used.

Another study using data from the French National Health Insurance also reported that TNF inhibitors were the most used biologics to initiate treatment (76.2%). The data from our study align with previous evidence, reflecting a clinical preference for TNF inhibitors, likely due to their availability and proven efficacy, experience, and safety (Pina Vegas et al., 2022).

When addressing the pharmacological management of PsA, it is important to consider treatment persistence, which is a critical factor reflecting efficacy, safety, satisfaction, and adherence. Persistence is defined as the time from initiation to discontinuation of the medication (Vegas et al., 2025; Cramer et al., 2008). It is considered high when it extends over long periods, which may vary depending on the study design, population and presence of risk factors for discontinuation of the biologic treatment, among other variables (Geale et al., 2020).

In our study, the median time patients persisted on biologic therapy was 35.5 months, with 60.6% of patients continuing with the same biologic they started treatment with. These results suggest high persistence in our population overall. Similarly, a study conducted in Spain found that 59.7% of PsA patients remained on their initial biologic for an average of 45.5 months (Cañete et al., 2020). And a study in France reported that 36% of patients persisted with the same biologic for an average of 36 months. However, it is noteworthy that persistence tends to decrease significantly over time (Vegas et al., 2025).

It is important to note that the relatively high persistence observed in our cohort may be partially explained by local contextual factors. In Colombia, biologic therapies are covered by the national health insurance system (PBS), minimizing out-of-pocket costs and potentially enhancing adherence. Furthermore, all patients in this study were managed at a high-complexity tertiary care center, where close and specialized follow-up may facilitate early identification of adverse events, better patient education, and timely treatment adjustments. These factors may contribute to prolonged treatment duration compared to reports from other settings.

The biologics with the highest persistence in our study were Secukinumab (65.2%) and Adalimumab (49%). These results align with previous findings from several studies and clinical trials, which have shown that Secukinumab and Adalimumab are the biologics with the highest persistence in PsA patients (Singh et al., 2018; Coates et al., 2025). In our cohort, a substantial proportion of patients who initially received a TNF inhibitor later switched to secukinumab, an IL-17 inhibitor. This aligns with international guidelines recommending a change in mechanism of action in cases of suboptimal response. This switching strategy may partly explain the high persistence observed with secukinumab (Carrascosa et al., 2022).

It is well known that for optimal management of PsA patients, therapy must be administered continuously (Walsh et al., 2018). In this study, only 5.5% of patients discontinued biologic therapy without changing or restarting it, while 12.6% stopped the treatment and then restarted it. The main reason for discontinuation of biologic therapy was adverse effects (34.8%). A study from United States showed 26.8% of the patients discontinued the treatment without changing or restarting it, and 5.8% interrupted the treatment and restarted the reference biologic drug (Walsh et al., 2018). Our study showed a lower rate of biologic therapy interruption and aligns with existing evidence, which states that biologic treatment is most often interrupted due to therapeutic failure or adverse events (Merola et al., 2017).

In our study, 39.4% of patients experienced a change in biologic medication, with an average time between the initial biologic and the first switch of 22 months. In contrast, a study conducted in the United States found that 22.9% of PsA patients switched to a different biologic, with a median time to switch of approximately 6 months (Walsh et al., 2018). Therefore, a significant proportion of patients in our cohort underwent treatment changes, biologic persistence—measured in months—was longer compared to other reports.

It is important to note that biologic therapies became available in Colombia at different times compared to other countries, which may have influenced both treatment exposure and clinical experience. For instance, our experience with golimumab is limited, likely due to its later regulatory approval compared to adalimumab, which may account for the shorter observed exposure time (Supplementary Material 3 provides details on INVIMA approval dates for the biologics used between 2011 and 2021).

Regarding medication changes, in our study, most of the patients with at least one biologic switch were receiving Adalimumab (40%), followed by Etanercept (30%). Secukinumab was the most frequently selected biologic for therapy modification, and by the end of follow-up, 31.7% of patients were on this medication. A study conducted in the United States found showed similar preferences who experienced at least one biologic switch, most were initially treated with Etanercept, followed by Adalimumab and the most common biologic to which patients switched was Adalimumab (48.4%) (Walsh et al., 2018).

The treatment of PsA is often complicated by its heterogeneous presentation and evolving clinical course, which frequently necessitates the use of adjunctive medications (Perrone et al., 2022). In our study, the most prescribed complementary therapies were conventional DMARDs (57.5%), methotrexate (44.1%), corticosteroids (27.6%), and NSAIDs (22.8%). These findings align with EULAR guidelines, which recommend NSAIDs and csDMARDs as complementary therapies alongside biologics (Gossec et al., 2024). Some experts suggest combining methotrexate with biologics may also reduce side effects by allowing lower doses of biologics (Merola et al., 2017; Gossec et al., 2024; Coates et al., 2016; Singh et al., 2018). Our findings regarding the use of complementary therapies such as methotrexate and corticosteroids align with recent studies showing that combination strategies may improve biologic retention and reduce immunogenicity (Scriffignano et al., 2023), underscoring the importance of individualized treatment approaches in real-world clinical practice.

In our study, 15.8% of patients experienced some adverse effect during the time they were on biologic treatment. The most common were infections (85%), mild skin allergic reactions (20%) and diarrhea (15%), consistent with meta-analyses showing infections as the most frequent adverse effect (Singh et al., 2011).

## Conclusion

Our population had a high persistence to biologic treatment, with only a small percentage of patients discontinued treatment permanently, mainly due to side effects. Biologic agents, primarily TNF and IL-17 inhibitors, are widely used in the management of PsA, with high treatment persistence, especially with Adalimumab and Secukinumab. There are few studies evaluating the PsA population treated with biologics in Latin America, so these findings provide unique data from a cohort of patients treated at a high-complexity hospital in Colombia, with a particular focus on persistence and biologic drug usage patterns. The findings highlight the heterogeneity of PsA, reinforcing the importance of personalized and continuous management, guided by specialists.

## Limitations

This study has several limitations inherent to its retrospective and observational design, which precludes the establishment of causal relationships. Data were obtained from electronic health records, and clinical information was not always systematically documented.

First, although all patients were evaluated by rheumatologists trained in psoriatic disease and classified according to CASPAR criteria, no formal dermatologic assessment was performed. Consequently, validated tools for assessing psoriasis severity, such as PASI or NAPSI, were not used.

Second, the absence of standardized clinimetric tools or imaging confirmation for enthesitis may have led to underreporting or misclassification of this domain, as well as of disease duration.

Finally, due to regulatory timelines and drug availability in Colombia, some biologic agents currently used in PsA management were not included in the study period.

## Data Availability

The raw data supporting the conclusions of this article will be made available by the authors, without undue reservation.

## Abbreviations

PsA, Psoriatic arthritis; TNF, tumor necrosis factor; IL, Interleukin; CASPAR, Classification Criteria for Psoriatic Arthritis; INVIMA, National Institute for Food and Drug Surveillance; JAK, Janus kinase; ICD-10, International Classification of Diseases; IBD, Inflammatory Bowel Disease; ESR, Erythrocyte sedimentation rate; PCR, C-reactive protein; MRI, Magnetic Resonance Imaging; ICU, intensive care unit; MOA, mechanism of action; PBS, Health Benefits Plan; EULAR, European League Against Rheumatism; NSAIDs, nonsteroidal anti-inflammatory drugs; csDMARDs, Disease-modifying antirheumatic drugs; PASI, Psoriasis Area and Severity Index; NAPSI, Nail Psoriasis Severity Index; BMI, body mass index; HLA-B27, human leukocyte antigen B27.
