# Supplementary material for: High persistence of biologic therapy in patients with Psoriatic arthritis: a real-world evidence from a high-complexity hospital in Colombia
Source: Front Pharmacol. 2025 Aug 29;16:1559168. doi: 10.3389/fphar.2025.1559168 (PMC12426402; doi:10.3389/fphar.2025.1559168)
Supplement: Supplementary file 1 [file DataSheet3.docx]

**Annex 3: INVIMA approval dates for biologicals used between 2011 and 2021.**

Biologic Trade name Year of approval (INVIMA) Mechanism of action

| Adalimumab | Humira® | 2007 | Anti-TNF |
| --- | --- | --- | --- |
| Etanercept | Enbrel® | 2005 | Anti-TNF |
| Golimumab | Simponi® | 2013 | Anti-TNF |
| Secukinumab | Cosentyx® | 2017 | Anti-IL-17A |
| Ustekinumab | Stelara® | 2012 | Anti-IL-12/23 |
| Guselkumab | Tremfya® | 2020 | Anti-IL-23 |
