# Supplementary material for: High persistence of biologic therapy in patients with Psoriatic arthritis: a real-world evidence from a high-complexity hospital in Colombia
Source: Front Pharmacol. 2025 Aug 29;16:1559168. doi: 10.3389/fphar.2025.1559168 (PMC12426402; doi:10.3389/fphar.2025.1559168)
Supplement: Supplementary file 2 [file DataSheet2.docx]

**Annex 2: Reasons for changes in Biological Therapy**

| **Reason for biologic change** | First biologic n= 50 | Second biologic n= 24 | Third biologic n= 10 |
| --- | --- | --- | --- |
| Lack of treatment response | 62,0 | 68,0 | 80,0 |
| Adverse effect | 12,0 | 20,0 | 10,0 |
| Administrative procedures | 6,0 | 0,0 | 10,0 |
| Other reason | 20,0 | 12,0 | 0,0 |
