# Supplementary material for: High persistence of biologic therapy in patients with Psoriatic arthritis: a real-world evidence from a high-complexity hospital in Colombia
Source: Front Pharmacol. 2025 Aug 29;16:1559168. doi: 10.3389/fphar.2025.1559168 (PMC12426402; doi:10.3389/fphar.2025.1559168)
Supplement: Supplementary file 3 [file DataSheet1.docx]

**Annex 1: Biologics Administered at Each Change in Patients with Psoriatic Arthritis**

| **Variable** | **N = 127** | | **n (%)** | | |
| --- | --- | --- | --- | --- | --- |
| **Biological Treatment** |  |  | | |  |
| **People with a biologic change** | 127 | 50 (39.4) | |  |  |
| **Number of changes** | 50 |  | |  |  |
| First biologic change |  | 26 (52.0) | |  |  |
| Second biologic change |  | 14 (28.0) | |  |  |
| Third biologic change |  | 10 (20.0) | |  |  |
| **Biologic used in first change** | 26 |  | |  |  |
| Secukinumab |  | 8 (30.8) | |  |  |
| Adalimumab |  | 5 (19.2) | |  |  |
| Golimumab |  | 4 (15.4) | |  |  |
| Tofacitinib |  | 4 (15.4) | |  |  |
| Ustekinumab |  | 3 (11.5) | |  |  |
| Ixekizumab |  | 1 (3.8) | |  |  |
| Guselkumab |  | 1 (3.8) | |  |  |
| **Biologic used in second change** | 14 |  | |  |  |
| Secukinumab |  | 5 (35.7) | |  |  |
| Etanercept |  | 4 (28.6) | |  |  |
| Golimumab |  | 2 (14.3) | |  |  |
| Ustekinumab |  | 2 (14.3) | |  |  |
| Adalimumab |  | 1 (7.1) | |  |  |
| **Biologic used in third change** | 10 |  | |  |  |
| Secukinumab |  | 4 (40.0) | |  |  |
| Etanercept |  | 2 (20.0) | |  |  |
| Guselkumab |  | 1 (10.0) | |  |  |
| Adalimumab |  | 1 (10.0) | |  |  |
| Ixekizumab |  | 1 (10.0) | |  |  |
| Tofacitinib |  | 1 (10.0) | |  |  |
| **Most recent biologic** | 105 |  | |  |  |
| Secukinumab |  | 31 (29.5) | |  |  |
| Adalimumab |  | 27 (25.7) | |  |  |
| Etanercept |  | 16 (15.2) | |  |  |
| Golimumab |  | 13 (12.4) | |  |  |
| Ustekinumab |  | 6 (5.7) | |  |  |
| Guselkumab |  | 5 (4.7) | |  |  |
| Tofacitinib |  | 4 (3.8) | |  |  |
| Ixekizumab |  | 2 (1.9) | |  |  |
| Certolizumab |  | 1 (0.95) | |  |  |
| **Additional treatment to biologic** | 127 |  | |  |  |
| Conventional DMARDs |  | 73 (57.5) | |  |  |
| Methotrexate |  | 56 (44.1) | |  |  |
| Steroids |  | 35 (27.6) | |  |  |
| NSAIDs |  | 29 (22.8) | |  |  |
| Leflunomide |  | 11 (8.6) | |  |  |
| Cyclosporine |  | 7 (5.5) | |  |  |
| Sulfasalazine |  | 6 (4.7) | |  |  |
| Chloroquine |  | 1 (0.8) | |  |  |
| Azathioprine |  | 1 (0.8) | |  |  |
| **Adverse effects** | 127 | 20 (15.8) | |  |  |
| Time between biologic administration and adverse effect** | 12 | 15.5 (37) | |  |  |
| **Adverse effects presented** | 20 |  | |  |  |
| Infection |  | 17 (85.0) | |  |  |
| Mild allergic skin reaction |  | 4 (20.0) | |  |  |
| Diarrhea |  | 3 (15.0) | |  |  |
| Thrombosis at venipuncture site |  | 2 (10.0) | |  |  |
| Herpes activation |  | 2 (10.0) | |  |  |
| Paradoxical reaction |  | 2 (10.0) | |  |  |
| Elevated transaminases |  | 1 (5.0) | |  |  |

Source: Author´s own elaboration.

** Mean (Standard Deviation)*  *** Median (Interquartile Range)*
